# Phonemic restoration in Alzheimer’s disease and semantic dementia: a preliminary investigation

**DOI:** 10.1093/braincomms/fcac118

**Published:** 2022-05-07

**Authors:** Jessica Jiang, Jeremy C. S. Johnson, Maï-Carmen Requena-Komuro, Elia Benhamou, Harri Sivasathiaseelan, Damion L. Sheppard, Anna Volkmer, Sebastian J. Crutch, Chris J. D. Hardy, Jason D Warren

**Affiliations:** Dementia Research Centre, Department of Neurodegenerative Disease, UCL Queen Square Institute of Neurology, London WC1N 3AR, UK

**Keywords:** phonemic restoration, semantic dementia, Alzheimer’s disease, speech perception, auditory processing

## Abstract

Phonemic restoration—perceiving speech sounds that are actually missing—is a fundamental perceptual process that ‘repairs’ interrupted spoken messages during noisy everyday listening. As a dynamic, integrative process, phonemic restoration is potentially affected by neurodegenerative pathologies, but this has not been clarified. Here, we studied this phenomenon in 5 patients with typical Alzheimer’s disease and 4 patients with semantic dementia, relative to 22 age-matched healthy controls. Participants heard isolated sounds, spoken real words and pseudowords in which noise bursts either overlaid a consonant or replaced it; a tendency to hear replaced (missing) speech sounds as present signified phonemic restoration. All groups perceived isolated noises normally and showed phonemic restoration of real words, most marked in Alzheimer’s patients. For pseudowords, healthy controls showed no phonemic restoration, while Alzheimer’s patients showed marked suppression of phonemic restoration and patients with semantic dementia contrastingly showed phonemic restoration comparable to real words. Our findings provide the first evidence that phonemic restoration is preserved or even enhanced in neurodegenerative diseases, with distinct syndromic profiles that may reflect the relative integrity of bottom-up phonological representation and top-down lexical disambiguation mechanisms in different diseases. This work has theoretical implications for predictive coding models of language and neurodegenerative disease and for understanding cognitive ‘repair’ processes in dementia. Future research should expand on these preliminary observations with larger cohorts.

## Teaser

Jiang *et al*. studied phonemic restoration of words in Alzheimer and semantic dementia patients versus healthy controls. All groups showed phonemic restoration of real words. Versus controls, phonemic restoration of pseudowords was enhanced in semantic dementia and reduced in Alzheimer’s disease. Findings suggest a retained perceptual repair mechanism in dementia.

## Graphical Abstract

**Graphical Abstract:**
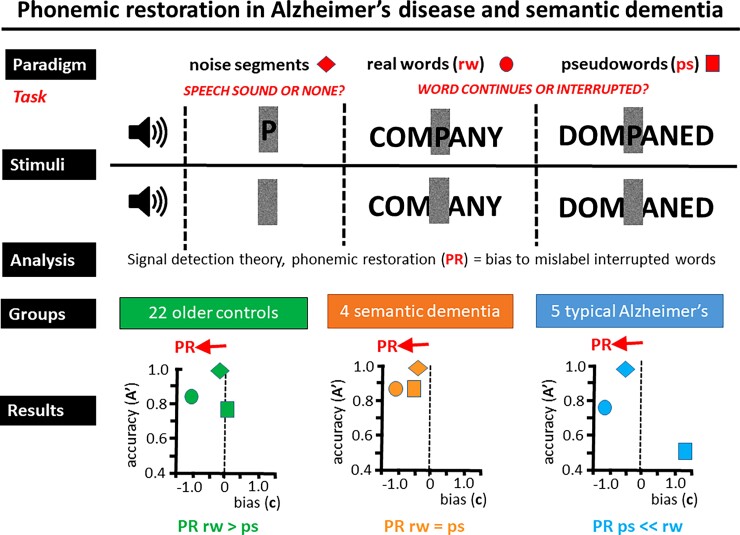

## Introduction

The speech we hear in daily life is often interrupted by external sounds, yet we generally perceive spoken messages as continuous and coherent. Our brains ‘repair’ interrupted messages by phonemic restoration: a fundamental physiological process whereby speech sounds that are obscured by noise are filled-in perceptually to reconstitute the underlying signal. Phonemes, the smallest units of spoken language, are constituted by specific combinations of acoustic spectrotemporal features that define them as a special class of auditory objects^1^; phonemic perception is, therefore, a touchstone for more fundamental mechanisms of auditory object processing. In the original experiment to address phonemic restoration, Warren^2^ observed that when a key phoneme was artificially excised from a spoken sentence, listeners were unable to identify the location of the missing phoneme when it was ‘filled-in’ with a coughing sound even though they could locate a corresponding silent gap accurately. This key result has since been replicated with a variety of interpolated noises.^3,4^

The original Warren paradigm was refined by Samuel in a series of experiments.^5–8^ Presentation of single words containing a white noise segment that either replaced a phoneme or was added to the phoneme allowed quantification of perceptual restoration using the framework of signal detection theory. This paradigm allowed an exploration of factors such as phonemic class and position, word frequency, duration and semantic predictability (real words versus pseudowords) and attentional set. Taken together, the findings from these experiments demonstrated that phonemic restoration depends on an adequate acoustic schema (incorporating a ‘speech-like’ noise) that is filled in according to expectations established by lexical context. In neural terms, the component processes of phonemic restoration are mediated by ‘bottom-up’ perceptual mechanisms (spectrotemporal featural synthesis and template matching) that parse incoming auditory signals and ‘top-down’ semantic mechanisms that predictively decode ambiguous signals based on stored knowledge of words.^9–14^ These mechanisms are computationally demanding and depend on synchronized activity across large-scale neural networks, encompassing posterior superior temporal and inferior frontal cortices of the dominant cerebral hemisphere.^9,13–15^

Phonemic restoration has been studied in certain clinical contexts. It appears to be unaffected by mild degrees of hearing loss^10^ and may be amplified in healthy older listeners, perhaps due to increased reliance on top-down lexical (rather than high-fidelity perceptual) mechanisms for processing speech signals.^12,16,17^ An increased tendency for phonemic restoration has also been found in developmental dyslexia,^18^ perhaps reflecting less stable acoustic phonological representations. On both physiological and neuroanatomical grounds, phonemic restoration is likely to be altered in neurodegenerative dementias, and the distinctive clinical and neuroanatomical profiles of these diseases predict differing consequences for perceptual restoration.^19^ Alzheimer’s disease is typically an amnestic clinical syndrome underpinned by degeneration of a temporo-parietal ‘default-mode’ network.^20,21^ It is associated with deficits of auditory scene analysis affecting sound segregation and streaming, spatial hearing and dichotic digit identification^22–27^ and impaired understanding of sinewave-degraded speech.^28^ Bottom-up processes of perceptual analysis supporting phonemic restoration are, therefore, likely to be affected in Alzheimer’s disease. In contrast, semantic dementia is characterized by impaired semantic memory due to selective degeneration of the anterior temporal cortex^29,30^; understanding of sinewave-degraded speech in semantic dementia is reduced for semantically unpredictable messages,^28^ suggesting that top-down mechanisms of phonemic restoration based on semantic disambiguation may be affected in this disease. These putatively distinct alterations of phonemic restoration in Alzheimer’s disease and semantic dementia might, therefore, be probed by varying the familiarity of the spoken word stimulus, thereby modulating the degree to which lexical recognition mechanisms are engaged. However, phonemic restoration has not previously been studied in neurodegenerative disease.

Here, we investigated phonemic restoration in patients with canonical syndromes of Alzheimer’s disease and semantic dementia in relation to healthy controls. We adapted the experimental paradigm described previously by Samuel^6^ and Del Tufo and Myers^18^ in which we used single real word and pseudoword stimuli containing a white noise segment that either replaced a phoneme or was added to the phoneme. We hypothesized that, in comparison to healthy controls, patients with Alzheimer’s disease would show increased phonemic restoration of real words but reduced restoration of pseudowords, due to impaired early perceptual analysis of phonemes with increased reliance on top-down processes of lexical recognition. In contrast, we hypothesized that patients with semantic dementia would show reduced phonemic restoration of both word classes, due to increased reliance on early perceptual mechanisms with attenuated top-down semantic influences on lexical processing.

## Materials and methods

### Participants

Four patients with semantic dementia and five with typical Alzheimer’s disease were recruited via a specialist cognitive clinic. All patients fulfilled consensus criteria with compatible brain MRI profiles for their diagnosis^29,31^ and had clinically mild-to-moderate disease. Twenty-two healthy control participants with no history of neurological or psychiatric disorders were recruited from the Dementia Research Centre database of healthy volunteers. No participant had abnormal peripheral hearing other than age-related hearing loss (see Supplementary Materials online for details of audiometry procedure) or significant cerebrovascular burden on MRI. All participants had a comprehensive general neuropsychological assessment (Table 1).

**Table 1:** General demographic, clinical and neuropsychological characteristics of participant groups

	Healthy controls	SD	AD	Omnibus significance test
Demographic and clinical				
Gender (F:M)	10:12	0:4	2:3	Fisher’s exact = 0.314
Age (years)	66.45 (6.34)	63.00 (8.33)	69.80 (7.95)	*F*(2,28) = 1.12; *P* = 0.342
Handedness (L:R)	2:18^a^	0:4	1:4	Fisher’s exact = 0.845
Education (years)	15.65 (2.74)^a^	15.00 (2.00)	16.00 (4.00)	*F*(2,26) = 0.14; *P* = 0.874
Symptom duration (years)	N/A	5.25 (2.22)	5.20 (2.17)	*t*(7) = 0.03; *P* = 0.974
Mean peripheral hearing score (best ear; dB)	17.75 (8.43)^b^	20.25 (8.54)	25.20 (10.03)	*F*(2,18) = 1.26; *P* = 0.308
General intellect				
MMSE (/30)	29.67 (0.65)^b^	**25.00** (**5.60)**	**25.20** (**3.83)**	*F*(2,18) = 6.11; *P* = 0.010
Episodic memory				
RMT words (/50)	47.95 (3.70)^c^	**35.50** (**6.61)**	**34.40** (**8.32)**	*F*(2,25) = 19.92; *P* < 0.001
RMT faces (/50)	42.47 (3.91)^c^	**32.00** (**4.55)**	**30.60** (**4.88)**	*F*(2,25) = 22.50, *P* < 0.001
Working memory				
Digit span forwards (max)	6.79 (1.03)^c^	7.50 (0.58)	6.60 (0.84)	*F*(2,25) = 0.94; *P* = 0.405
Digit span backwards (max)	5.63 (1.34)^c^	5.50 (1.91)	4.60 (0.55)	*F*(2,25) = 1.19; *P* = 0.322
Executive functions				
Stroop suppression (s)	55.68 (11.12)^c^	**87.33** (**13.61)**^d^	**135.00 (41.75)** ^d,e^	*F*(2,23) = 31.79; *P* < 0.001
Letter fluency (total)	17.78 (6.83)^f^	10.00 (8.19)^d^	12.60 (3.85)	*F*(2,23) = 2.59; *P* = 0.097
Category fluency (total)	24.94 (7.03)^f^	**5.75** (**6.85)**^d^	**13.40** (**6.23)**	*F*(2,24) = 15.52; *P* < 0.001
Language skills				
GNT (/30)	26.05 (2.37)	**3.75** (**7.5)**^g^	**16.80** (**8.34)**	*F*(2,25) = 40.01; *P* < 0.001
BPVS (/150)	147.63 (2.22)^c^	**82.50** (**65.76)**^g^	146.40 (2.07)	*F*(2,25) = 13.80; *P* < 0.001
Posterior cortical functions				
Arithmetic (/24)	16.05 (4.82)^c^	15.50 (4.20)	**7.25 (4.57)** ^d,e^	*F*(2,24) = 5.82; *P* = 0.009
VOSP object decision (/20)	19.05 (1.43)^c^	17.67 (1.53)^d^	**15.60** (**2.61)**	*F*(2,24) = 8.44; *P* = 0.002

Mean (standard deviation) values are given for continuous variables; counts are given for categorical variables (maximum scores for neuropsychological tests are indicated in parentheses where appropriate). Significantly worse performance compared with the healthy control group is indicated in bold. AD, patient group with Alzheimer’s disease; BPVS, British Picture Vocabulary Scale; F, female; GNT, Graded Naming Test; L, left; MMSE, Mini-Mental Stat Examination; N/A, not applicable; R, right; RMT, Recognition Memory Test; SD, patient group with semantic dementia; VOSP, visual object space perception. Missing data are indicated below.

^a^ Data missing for two participants.

^b^ Data missing for 10 participants.

^c^ Data missing for three participants.

^d^ Data missing for one participant.

^e^ Significantly worse than the SD group.

^f^ Data missing for four participants.

^g^ Significantly worse than the AD group.

All participants gave informed consent to take part in the study. Ethical approval was granted by the UCL-NHNN Joint Research Ethics Committees, in accordance with the Declaration of Helsinki guidelines.

### Experimental stimuli

**Figure 1:**
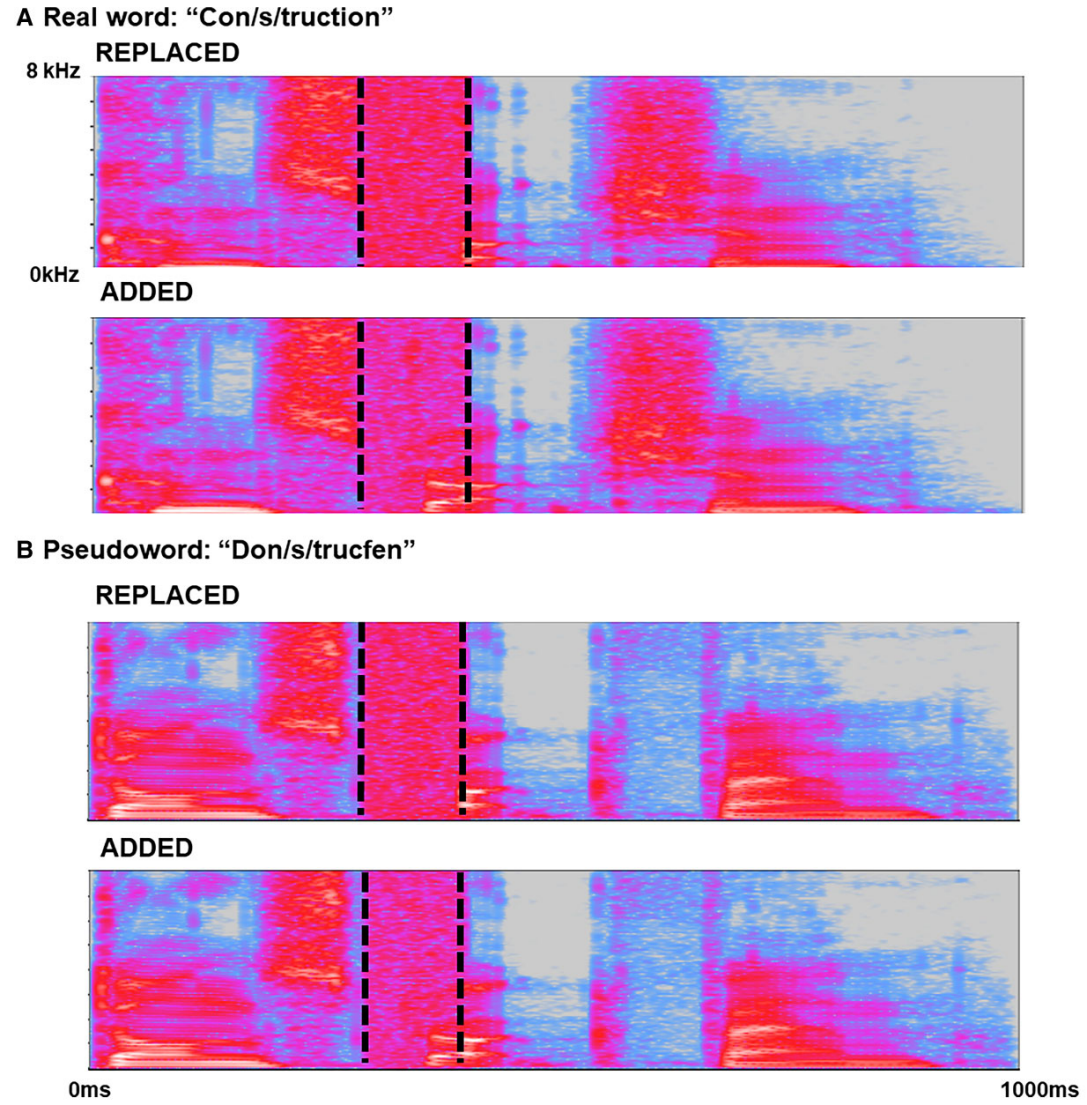
**Representative time–frequency spectrograms of stimuli for the different experimental conditions based on word carriers.** The *y*-axis of each spectrogram codes frequency (kilohertz); the *x*-axis codes time (milliseconds). In all example spectrograms, vertical dotted lines show the boundaries of the critical (target) spoken phoneme (indicated in the word heading each panel); the spoken word segment containing the target phoneme has been manipulated in each case with white noise. (**A**) Example stimuli based on real word carriers; (**B**) stimuli based on pseudoword carriers. In each panel, an example of a ‘Replaced’ stimulus (i.e. white noise replacing the spoken consonant) is shown above and an example of an ‘Added’ stimulus (i.e. white noise superimposed over the spoken consonant) is shown below. Spectrograms were generated in Audacity v 3.0.0 (https://audacityteam.org).

Forty tri-syllabic words and 40 matched, phonetically plausible pseudowords (created by changing specific phonemes in each real word) were recorded by a male native English speaker with a Standard Southern British English accent (stimulus lists in Supplementary Table 1, recording details and example sound files in Supplementary Material online). Recordings were edited by inserting a white noise segment; in half the recordings, the noise was added to a consonant (e.g. real word, A/*PP*/EARANCE; pseudoword, I/*PP*/EAGANCE), while in the remainder, noise replaced the consonant completely (e.g. A/**__**/EARANCE or I/**__**/EAGANCE). This manipulation yielded a total of four-word stimulus conditions (two carrier conditions: Real words/Pseudowords) × (two noise conditions: Replaced/Added), each comprising 40 trials (Fig. 1).

Separately, we created a perceptual control stimulus set to assess participants’ ability to discriminate ‘Replaced’ versus ‘Added’ stimuli acoustically. Control stimuli comprised 40 isolated noise segments, 20 in which the noise was superimposed on a spoken consonant (e.g. ‘***_****S**_***’; equivalent to ‘Added’ noise segments in spoken words) and 20 without an associated speech sound (‘**__**’; equivalent to ‘Replaced’ noise segments in spoken words).

Further details are available in Supplementary Materials online.

### Experimental procedures

All testing sessions took place in a quiet room and stimuli were administered through MATLAB on a Windows laptop, via headphones (Audio-Technica ATH-M50x) set at a comfortable listening level volume (at least 70 dB). During the experimental sessions, no feedback on performance was given and no time limits were imposed.

### Perceptual control task on isolated noise segments

The perceptual control stimuli (noise segments) were presented in a randomized order as a single block of 40 trials. Participants were told that they would hear a series of noises and their task on each trial was to decide whether it was ‘only noise’ or ‘noise-plus-speech’.

### Real word and pseudoword conditions

Adapting a previous procedure,^18^ both ‘Added’ and ‘Replaced’ stimuli were split into 4 blocks of 40 trials, each containing 10 trials from each stimulus condition. Trials were randomized within each block; ‘Added’ and ‘Replaced’ versions of the same word and matched pseudowords never occurred in the same block.

Participants were told that they would hear a series of words (either ‘real’ or ‘made-up’) containing a noise and asked on each trial to determine whether the word continued through the noise (‘Added’) or was interrupted (‘Replaced’). Participants were first familiarized with the stimuli and the task to ensure understanding. Pictorial cue cards (Supplementary Fig. 1) were provided as aids during the experimental session; participants could respond verbally or by pointing to the cue card.

### Statistical analysis

Data were analysed using STATAv14, unless specified. Between-group comparisons of continuous demographic and neuropsychological data used ANOVA. Comparisons for categorical data used Fisher’s exact test.

We used the non-parametric *A′* as a measure of the sensitivity of discrimination between ‘Added’ and ‘Replaced’ stimuli (see the rationale in Supplementary material)^32^ and the criterion location ***c*** as our measure of bias in participants’ responses. For each participant, *A′* and ***c*** were calculated for each experimental condition separately using an Excel Workbook.^33^ Values of *A′* can range from 0 to 1: an *A′* of 1 would indicate perfect discrimination (i.e. no phonemic restoration). An *A′* of 0.5 would indicate that ‘Added’ and ‘Replaced’ versions of the presented words were indistinguishable; this could indicate either all words labelled as ‘Added’ (i.e. complete phonemic restoration) or all words labelled as ‘Replaced’ (no phonemic restoration) or some combination of ‘Replaced’/‘Added’ confusions. *A′* < 0.5 would indicate a tendency to select the response ‘opposite’ to that defined as a ‘hit’ (e.g. a tendency to report ‘Added’ words as ‘Replaced’ and vice versa).

*A′* values can be further interpreted by examining the direction of ***c***: negative values of ***c*** indicate a bias towards responding ‘Added’ over ‘Replaced’ (i.e. phonemic restoration occurred in the stimuli) while positive values indicate a bias towards responding ‘Replaced’ over ‘Added’. Values near zero indicate no particular bias towards one response category over the other.

**Figure 2:**
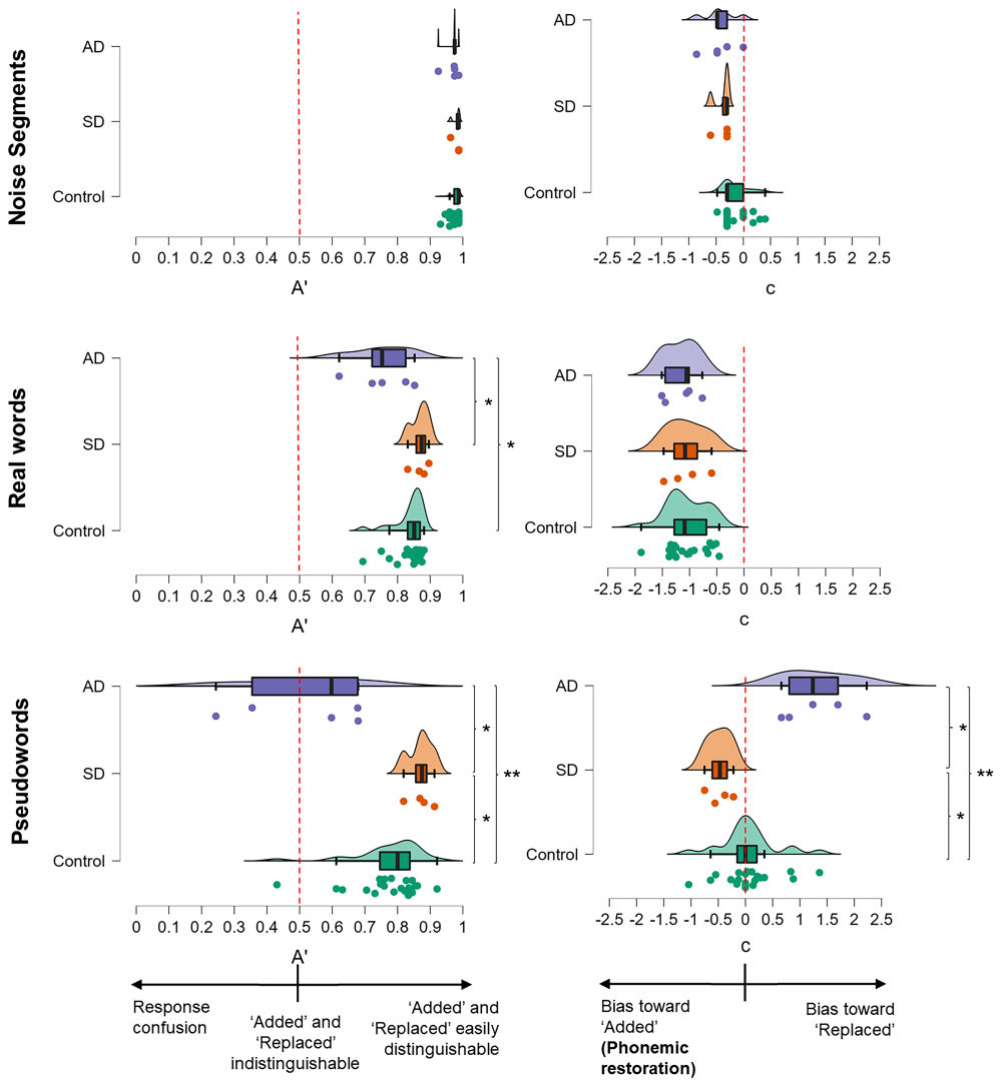
**Summary of participant group profiles for response sensitivity and bias across experimental conditions.** Raincloud plots of individual data for response sensitivity (left panels) and bias (right panels) for all experimental conditions and participant groups. Boxes represent the interquartile range, and whiskers indicate the overall range of values in each group; the vertical line in each box represents the median. The filled circles code values for individual participants. Sensitivity (*A*′) values typically lie between 0.5 (indicating the participant was unable to discriminate between ‘Replaced’ and ‘Added’ stimuli) and 1 (indicating perfect discrimination); values below 0.5 indicate response confusion (see text). For the measure of bias or criterion location (***c***), negative values indicate bias towards responding ‘Added’ (i.e. phonemic restoration) while positive values indicate bias towards responding ‘Replaced’. AD, participant group with Alzheimer’s disease; Control, healthy control participant group; Noise, perceptual control condition (isolated noise segments); Pseud, pseudoword experimental condition; Real, real word experimental condition; SD, participant group with semantic dementia. *Significant at *P* < 0.05; **significant at *P* < 0.01. For real worlds, *A′* differed significantly across groups [Kruskal–Wallis, χ^2^(2) = 9.24, *P* = 0.010], with the AD group having significantly lower median *A′* than healthy controls (Wilcoxon rank-sum, *z* = −2.22, *P* = 0.027) and the SD group (Wilcoxon rank-sum, z = −2.21, *P* = 0.028). ***c*** did not differ significantly across groups for real words [Kruskal–Wallis, χ^2^(2) = 0.79, *P* = 0.675]. For pseudowords, *A′* differed significantly across groups [Kruskal–Wallis, χ^2^(2) = 13.38, *P* = 0.001], with the AD group having significantly lower median *A′* than healthy controls (Wilcoxon rank-sum, *z* = −3.00, *P* = 0.003) and SD patients (Wilcoxon rank-sum, *z* = 2.45, *P* = 0.014), while the semantic dementia group had significantly higher *A’* than healthy controls (Wilcoxon rank-sum, z = 2.20, *P* = 0.03). ***c*** also differed significantly across groups for pseudowords [Kruskal–Wallis, χ^2^(2) = 14.11, *P* < 0.001], with significant differences between AD and controls (Wilcoxon rank-sum z = −3.00, *P* = 0.003); SDs and controls (Wilcoxon rank-sum z = 2.42, *P* = 0.016); and SDs and ADs (z = 2.45, *P* = 0.014).

Given the disparate group sizes, we used the non-parametric Kruskal–Wallis test to assess whether there was an effect of each separate diagnostic group on *A’* or ***c*** in each experimental condition. Where this omnibus test was significant, we conducted *post hoc* two-tailed Wilcoxon rank-sum tests to compare groups directly, to understand the direction of the effect. In addition, we generated difference scores for *A′* and ***c*** for the pseudoword and real word carrier conditions; this was also analysed as a dependent variable, using the framework described above. Finally, within each diagnostic group, we compared *A′* and ***c*** between carrier conditions directly using Friedman’s tests, in JASP v15. This software was also used to generate the raincloud plots^34^ in Fig. 2. Two-tailed tests were used for all analyses. No multiple comparisons correction was conducted due to the small sample size and the nature of the tests not being purely independent observations from one another.

To characterize the consistency and variability of individual patient performance profiles relative to healthy controls, we calculated the 5th and 95th percentiles for the healthy control group and identified patients in each dementia group who performed below the control 5th percentile or above the control 95th percentile.

### Data availability

The data that support the findings of this study are available on request from the corresponding author. The data are not publicly available because they contain information that could compromise the privacy of research participants.

## Results

Sensitivity (*A′*) and bias (***c***) values for all experimental conditions in each participant group are presented in Table 2 and Fig. 2. Table 3 shows individual raw scores in each experimental test and condition.

**Table 2:** Summary of participant group performance on phonemic restoration conditions

	Healthy controls		SD		AD	
Carrier condition	*A*′	*c*	*A*′	*c*	*A*′	*c*
Isolated noise segments	0.98 (0.02)	−0.16 (0.24)	0.98 (0.01)	−0.37 (0.15)	0.97 (0.02)	−**0.42** (**0.31)**
Words	0.84 (0.05)	−1.04 (0.36)	0.87 (0.03)^a^	−1.06 (0.38)	**0.75** (**0.09)**	−1.16 (0.31)
Pseudowords	0.77 (0.11)	0.06 (0.51)	**0.87** (**0.04)**^a^	−**0.48** (**0.23)**^a^	**0.51** (**0.20)**	**1.33** (**0.65)**
Difference between real words and pseudowords	0.07 (0.10)	−1.10 (0.63)	−0.00 (0.02)^a^	−0.58 (0.35)	**0.24** (**0.20)**	−**2.48** (**0.81)**^a^

Mean (standard deviation) phonemic restoration measures of sensitivity (*A’*) and bias (*c*) are shown for each participant group and word/sound condition. *A*′ values typically lie between 0.5 (indicating the participant was unable to discriminate between ‘Replaced’ and ‘Added’ stimuli) and 1 (indicating perfect discrimination); values below 0.5 indicate response confusion (see text). For the measure of bias or criterion location [*c*], negative values indicate bias towards responding ‘Added’ (i.e. phonemic restoration) while positive values indicate bias towards responding ‘Replaced’. Significant performance differences with respect to the healthy control group are indicated in bold. AD, patient group with Alzheimer’s disease; SD, patient group with semantic dementia.

^a^ Significantly different from the AD group.

**Table 3:** Individual raw scores across experimental conditions

	Control																							SD					AD					
	**1**	**2**	**3**	**4**	**5**	**6**	**7**	**8**	**9**	**10**	**11**	**12**	**13**	**14**	**15**	**16**	**17**	**18**	**19**	**20**	**21**	**22**	**Mean (SD)**	**23**	**24**	**25**	**26**	**Mean (SD)**	**27**	**28**	**29**	**30**	**31**	**Mean (SD)**
Isolated noise segments (/40)																																		
A|A	20	20	16	20	20	20	20	19	17	20	20	19	20	20	20	18	20	18	19	19	20	20	**19.3** (**1.1)**	20	20	20	20	**20.0** (**0.0)**	20	20	19	20	20	**19.8** (**0.4)**
R|A	1	1	1	1	2	1	1	1	1	1	1	1	1	1	1	1	1	1	2	1	1	1	**1.1** (**0.3)**	3	1	1	1	**1.5** (**1.0)**	6	2	1	1	2	**2.4** (**2.1)**
R|R	19	19	19	19	18	19	19	19	19	19	19	19	19	19	19	19	19	19	18	19	19	19	**18.9** (**0.3)**	17	19	19	19	**18.5** (**1.0)**	14	18	19	19	18	**17.6** (**2.1)**
A|R	0	0	4	0	0	0	0	1	3	0	0	1	0	0	0	2	0	2	1	1	0	0	**0.7** (**1.1)**	0	0	0	0	**0.0** (**1.0)**	0	0	1	0	0	**0.2** (**0.4)**
*A*′	0.99	0.99	0.93	0.99	0.98	0.99	0.99	0.97	0.95	0.99	0.99	0.97	0.99	0.99	0.99	0.96	0.99	0.96	0.96	0.97	0.99	0.99	**0.98** (**0.02)**	0.96	0.99	0.99	0.99	**0.98**(**0.01)**	0.93^a^	0.98	0.97	0.99	0.98	**0.97** (**0.02)**
Real words (/80)																																		
A|A	40	40	40	37	40	34	39	39	35	39	40	39	40	40	39	33	40	36	40	40	36	38	**38.4** (**2.2)**	40	38	40	39	**39.3** (**1.0)**	37	39	39	40	33	**37.6** (**2.8)**
R|A	21	21	36	19	22	18	22	25	24	18	20	20	21	24	21	25	23	16	24	20	18	15	**21.5** (**4.3)**	27	13	19	19	**19.5** (**5.7)**	30	33	21	28	29	**28.2** (**4.4)**
R|R	19	19	4	21	18	22	18	15	16	22	20	20	19	16	19	15	17	24	16	20	22	25	**18.5** (**4.3)**	13	27	21	21	**20.5** (**5.7)**	10	7	19	12	11	**11.8** (**4.4)**
A|R	0	0	0	3	0	6	1	1	5	1	0	1	0	0	1	7	0	4	0	0	4	2	**1.6** (**2.2)**	0	2	0	1	**0.8** (**1.0)**	3	1	1	0	7	**2.4** (**2.8)**
*A*′	0.87	0.87	0.78	0.84	0.86	0.80	0.85	0.82	0.75	0.87	0.88	0.86	0.87	0.85	0.85	0.69	0.86	0.85	0.85	0.88	0.83	0.88	**0.84** (**0.05)**	0.83	0.90^b^	0.88^b^	0.87	**0.87** (**0.03)**	0.72	0.75	0.85	0.83	0.62^a^	**0.75** (**0.09)**
Pseudowords (/80)																																		
A|A	28	36	25	14	27	26	29	39	27	33	32	26	25	25	36	25	3	31	26	15	30	32	**26.8** (**8.0)**	37	36	36	37	**36.5** (**0.6)**	3	10	14	0	3	**6.0** (**5.8)**
R|A	9	17	20	4	5	7	19	22	13	4	11	14	9	11	20	19	4	9	13	3	10	12	**11.6** (**5.9)**	21	12	8	15	**14.0** (**5.5)**	1	7	7	1	6	**4.4** (**3.1)**
R|R	31	23	20	36	35	33	21	18	28	36	29	26	31	29	20	21	36	31	27	37	30	28	**28.4** (**5.9)**	19	28	32	25	**26.0** (**5.5)**	39	33	33	39	34	**35.6** (**3.1)**
A|R	12	4	15	26	13	14	11	1	13	7	8	14	15	15	4	15	37	9	14	25	10	8	**13.2** (**8.0)**	3	4	4	3	**3.5** (**0.6)**	37	30	26	40	37	**34.0** (**5.8)**
*A*′	0.82	0.84	0.61	0.75	0.86	0.86	0.71	0.85	0.76	0.92	0.85	0.73	0.79	0.76	0.81	0.63	0.43	0.85	0.75	0.78	0.83	0.83	**0.77** (**0.11)**	0.82	0.88^b^	0.91^b^	0.87^b^	**0.87** (**0.04)**	0.68	0.60	0.68	0.24^a^	0.36^a^	**0.51** (**0.20)**

The table shows individual participant response totals in each of the main experimental conditions. Note that 20 trials of each stimulus type (Added/Replaced) were presented for each isolated noise segment condition and 40 trials for each word condition; the maximum score in each cell is therefore 20 for segments and 40 for real words/pseudowords. Stimulus conditions were delivered in a randomized order during the experimental session. A|A denotes that the participant correctly identified an ‘Added’ stimulus as ‘Added’; R|A denotes that the participant incorrectly identified a ‘Replaced’ stimulus as ‘Added’ (i.e. phonemic restoration); R|R denotes that the participant correctly identified a ‘Replaced’ stimulus as ‘Replaced’; A|R denotes that the participant incorrectly identified an ‘Added’ stimulus as ‘Replaced’.

^a^ Individual patients whose performance fell below the 5th percentile for the healthy control group.

^b^ Individual patients whose performance fell above the 95th percentile for the healthy control group.

### General characteristics of participant groups

Participant groups did not differ significantly in gender distribution, age, handedness, years of education or peripheral hearing function (all *P* > 0.05, Table 1). Patient groups did not differ in the mean symptom duration (*P* = 0.974). General neuropsychological profiles were in keeping with the syndromic diagnosis for each patient group (Table 1).

### Perceptual control task on isolated noise segments

*A′* did not differ significantly across diagnoses [χ^2^(2) = 1.79, *P* = 0.408]. *A′* was uniformly high across participant groups, indicating that ‘Added’ and ‘Replaced’ noise segments were easily discriminable acoustically (Fig. 2; Table 2).

### Word conditions

#### Real words

The effect of diagnosis on *A′* was significant [χ^2^(2) = 7.48, *P* = 0.023]. The Alzheimer’s disease group had significantly lower median *A′* than both healthy controls (*z* = −2.22, *P* = 0.027) and the semantic dementia group (*z* = −2.21, *P* = 0.028); there was no significant difference between the control and semantic dementia groups (*z* = −1.42, *P* = 0.155).

All groups showed a clear bias (***c***) towards reporting words as ‘Added’ rather than ‘Replaced’. The effect of diagnosis on ***c*** was not significant [χ^2^(2) = 0.79, *P* = 0.675].

#### Pseudowords

There was a significant effect of diagnosis on *A′*[χ^2^(2) = 13.38, *P* = 0.001]. The Alzheimer’s disease group had significantly lower median *A′* than both healthy controls (*z* = −3.00, *P* = 0.003) and the semantic dementia group (*z* = −2.45, *P* = 0.014), performing essentially at chance; the semantic dementia group had significantly higher *A′* than the healthy control group (*z* = 2.20, *P* = 0.03).

There was a significant effect of diagnosis on ***c*** [*χ*^2^(2) = 14.11, *P* < 0.001]. Healthy controls showed essentially no bias (***c***) in reporting pseudowords. Compared with healthy controls, the Alzheimer’s disease group showed a significantly greater bias towards reporting pseudowords as ‘Replaced’ (*z* = −3.00, *P* = 0.003), while the semantic dementia group showed a significantly greater bias towards reporting pseudowords as ‘Added’ (*z* = 2.42, *P* = 0.016).

### Comparisons between carrier conditions

The value of *A′* differed between real word and pseudoword conditions according to diagnosis [significant overall effect of diagnosis, χ^2^(2) = 9.24, *P* = 0.010]. This was driven by a greater difference between word conditions in the Alzheimer’s disease group than the healthy control (*z* = 2.43, *P* = 0.015) or semantic dementia groups (*z* = 2.45, *P* = 0.014); performance of the semantic dementia and healthy control groups did not differ significantly (*z* = 1.64, *P* = 0.102).

The value of ***c*** also differed between real word and pseudoword conditions according to diagnosis [χ^2^(2) = 12.09, *P* = 0.002]. This was driven by a greater difference between word conditions in the Alzheimer’s disease group than the healthy control (*z* = 3.06, *P* = 0.002) or semantic dementia groups (*z* = 2.45, *P* = 0.014); response bias in the semantic dementia and healthy control groups did no differ significantly (*z* = −1.64, *P* = 0.102).

When conditions were compared within diagnostic groups, all groups showed significantly lower *A′* both for real words [controls, *χ*^2^(1) = 22.00, *P* < 0.001; semantic dementia, *χ*^2^(1) = 4.00, *P* = 0.046; Alzheimer’s disease, *χ*^2^(1) = 5.00, *P* = 0.025] and pseudowords [controls, χ^2^(1) = 22.00, *P* < 0.001; semantic dementia, χ^2^(1) = 4.00, *P* = 0.046; Alzheimer’s disease, χ^2^(1) = 5.00, *P* = 0.025] than isolated noise segments. *A′* was significantly lower for pseudowords than real words in the healthy control group [χ^2^(1) = 4.55, *P* = 0.033] and Alzheimer’s disease group [χ^2^(1) = 5.00, *P* = 0.025] but not the semantic dementia group [χ^2^(1) = 0.00, *P* = 1.00].

All groups also showed significantly lower ***c*** (i.e. a bias towards reporting ‘Added’) for real words compared with isolated noise segments [controls, *χ*^2^(1) = 22.00, *P* < 0.001; semantic dementia, *χ*^2^(1) = 4.00, *P* = 0.025; Alzheimer’s disease, *χ*^2^(1) = 5.00, *P* = 0.025]. The Alzheimer’s disease group showed significantly higher ***c*** (i.e. a bias towards reporting ‘Replaced’) for pseudowords than isolated noise segments [*χ*^2^(1) = 5.00, *P* = 0.025]; response bias did not differ between the pseudoword and noise segment conditions in healthy controls [*χ*^2^(1) = 1.64, *P* = 0.201] or the semantic dementia group [*χ*^2^(1) = 4.00, *P* = 0.046]. All groups showed significantly lower ***c*** for real words than pseudowords [controls, *χ*^2^(1) = 22.00, *P* < 0.001; semantic dementia, *χ*^2^(1) = 4.00, *P* = 0.046; Alzheimer’s disease, χ^2^(1) = 5.00, *P* = 0.025].

### Individual patient performance profiles

For the isolated noise segment condition, one patient in the Alzheimer’s disease group had an *A*′ value below the healthy control 5th percentile.

For the real word condition, two patients with semantic dementia (50% of the group) had *A*′ values above the healthy control 95th percentile; whilst one patient with Alzheimer’s disease had an *A*′ value below the control 5th percentile.

For the pseudoword condition, three patients with semantic dementia (75% of the group) had *A*′ values above the healthy control 95th percentile, whilst two patients with Alzheimer’s disease (40% of the group) had *A*′ values below the control 5th percentile.

## Discussion

Here, we have presented evidence for phonemic restoration in two major dementias. Both the patient groups and healthy older listeners were highly accurate in discriminating whether or not isolated noise segments contained speech sounds. The less accurate performance across groups in the word conditions is unlikely simply to have reflected spectrotemporal feature discriminability (since spectrotemporal features were similar for the isolated noise segment and lexical conditions) or the proximity of additional spectrotemporal information surrounding the ‘target’ segment (since performance also differed between the real word and pseudoword conditions). The performance profile of the healthy control group here demonstrates phonemic restoration relatively greater for real spoken words than for pseudowords (as evidenced by a bias towards hearing interpolating noise bursts as overlaying rather than interrupting spoken words). This profile of retained phonemic restoration modulated by top-down lexical context effects is in line both with prevailing models of auditory word processing^35^ and with previous work in older listeners using alternative phonemic restoration paradigms.^12,16,17^ However, the nature and extent of phonemic restoration differed for the dementia groups. Whereas all participant groups showed evidence of phonemic restoration for real words, this was more marked in patients with Alzheimer’s disease than in healthy controls or patients with semantic dementia. Group profiles differed more substantially for phonemic restoration of pseudowords: here, healthy controls showed less accurate discrimination between noise conditions than for real words but no clear tendency towards phonemic restoration, while patients with Alzheimer’s disease showed a marked tendency towards perceiving noise segments as replacing phonemes (i.e. ‘suppression’ of phonemic restoration). In contrast, patients with semantic dementia performed comparably on discrimination of noise conditions in both pseudowords and real words, and indeed performed more accurately for pseudowords than did healthy controls.

The performance profiles in these dementia syndromes illuminate the underlying brain mechanisms of phonemic restoration. Taken together, the findings in Alzheimer’s disease and semantic dementia are consistent with a phonemic restoration model in which phonological representations (likely instantiated in the posterior superior temporal cortex) interact with a modulatory, top-down mechanism of semantic prediction and disambiguation (likely mediated by more anterior cortical regions). In the healthy brain, the interaction of phonological and semantic mechanisms primes ‘repair’ of real words over pseudowords.^6^ In Alzheimer’s disease, however, phonemic representations are damaged as part of a more general impairment of auditory object parsing,^24,26^ whereas the top-down semantic mechanism-mediating lexical recognition is less impaired: in this situation, an overriding effect of lexical prediction would tend to strongly promote ‘repair’ of real words and rejection of pseudowords. Contrastingly, in semantic dementia, the balance of phonological and semantic effects is reversed: here, the semantic disadvantage of pseudowords relative to real words is attenuated, since the damaged lexical predictive mechanism does not override intact bottom-up phonological processing.

Phonemic restoration could *a priori* be achieved by matching incoming speech signals to a stored lexical ‘template’ or through Gestalt continuity (the sensory expectation that phonological patterns corresponding to words tend to be spectrotemporally continuous).^15^ However, the strong word category effect in the Alzheimer’s disease group here suggests that top-down lexical template matching plays a dominant role in phonemic restoration. The comparable performance in the real word and pseudoword conditions in the semantic dementia group corroborates this interpretation. Whereas perceptual completion of real words might be based on general lexical familiarity, ‘rejection’ of pseudowords (here A/R errors, reduced relative to healthy controls in the semantic dement group; Table 3) depends on a more fine-grained semantic computation, corresponding operationally to a ‘search’ of the stored semantic lexicon. If this process is deficient (as in semantic dementia), then pseudoword processing becomes relatively more dependent on—still intact—bottom-up perceptual processing (so A/R errors are less likely). The profile observed in the semantic dementia group here further suggests that lexical predictive decoding is normally mediated by the anterior temporal cortex (the core locus of damage in semantic dementia). While neuroanatomical models of phonemic restoration have not foregrounded this brain region,^9,15^ it has been implicated in predictive decoding of word identity.^36^

We regard this study as a preliminary investigation to motivate and inform further work to more fully characterize the processes of phonemic restoration in neurodegenerative disease. Our study has a number of limitations and raises important issues that need clarification. The nature of phonemic restoration in healthy older listeners and the factors that influence this have not yet been defined fully: this requires systematic evaluation. For example, stimulus properties such as manner of articulation and placement of target phonemes might affect phonemic restoration^6^ and might interact with peripheral hearing function, attention and other cognitive processes that are potentially altered in normal ageing. Further, our small patient cohort size is likely to have limited power to detect disease effects; on the other hand, performance varied widely among individual patients (and indeed, among healthy control participants; see Fig. 2 and Table 3), implying that any generalization to group-level signatures should be cautious. This study does not allow a determination of the individual characteristics that may potentially have driven this variability. It does not appear simply to reflect disease stage or severity, acknowledging that any such measure is problematic in these syndromes, particularly if applied across diseases. A related issue concerns retained vocabulary in patients with semantic dementia: it is unlikely that comprehension of the real word list was affected uniformly among the patients with semantic dementia studied here and varying levels of lexical-semantic decoding may have influenced individual patient profiles. However, the use of personalized stimulus lists would greatly complicate the interpretation of disease group profiles of phonemic restoration, as these stimuli would also vary widely in acoustic characteristics. More fundamentally, thresholds for the perception of speech in noise as well as executive processes that guide perceptual decisions on degraded speech signals are likely to vary in dementia and between different neurodegenerative disorders^28,37^: a complete picture of phonemic restoration in these diseases will entail a better understanding of these processes.

These caveats notwithstanding, it is remarkable that a neural mechanism for ‘repairing’ degraded speech can be preserved or relatively enhanced in these neurodegenerative diseases, and further, stratifies different pathologies. We propose that the striking polarity of phonemic restoration effects between real words versus pseudowords in the Alzheimer’s disease group reflects a compensatory mechanism that tends to maintain the intelligibility of speech despite impaired auditory scene processing.^22–27^ Future work should test this hypothesis and extend the present findings to larger and more diverse patient cohorts, addressing the limits and influences on phonemic restoration in neurodegenerative disease and establishing its neural basis using functional neuroimaging. Dynamic neuroanatomical techniques such as magnetoencephalography with exquisite temporal resolution could potentially dissect the time courses of the component neural mechanisms that underpin phonemic restoration and reveal how these mechanisms contribute to a final percept and behavioural decision. As disease-modifying therapies for dementia become feasible, there is an urgent need to harness dynamic and fundamental neurophysiological processes—such as phonemic restoration—that could be targets for intervention and provide a rapid readout of therapeutic effects on neural circuit function.

## Supplementary Material

fcac118_Supplementary_Data
