# Supplementary material for: Phonemic restoration in Alzheimer’s disease and semantic dementia: a preliminary investigation
Source: Brain Commun. 2022 May 7;4(3):fcac118. doi: 10.1093/braincomms/fcac118 (PMC9123842; doi:10.1093/braincomms/fcac118)
Supplement: fcac118_Supplementary_Data [file fcac118_supplementary_data.zip › Jiang_SupplementarySoundfilesLegend.docx]

Supplementary Soundfiles Legend

1. Jiang_Soundfile_SegmentAdded
2. Jiang_Soundfile_SegmentReplaced
3. Jiang_Soundfile_WordAdded
4. Jiang_Soundfile_WordReplaced
5. Jiang_Soundfile_PseudowordAdded
6. Jiang_Soundfile_PseudowordReplaced
