# Supplementary material for: Phonemic restoration in Alzheimer’s disease and semantic dementia: a preliminary investigation
Source: Brain Commun. 2022 May 7;4(3):fcac118. doi: 10.1093/braincomms/fcac118 (PMC9123842; doi:10.1093/braincomms/fcac118)
Supplement: fcac118_Supplementary_Data [file fcac118_supplementary_data.zip › Supplementary_material.docx]

**SUPPLEMENTARY MATERIAL**

**Phonemic restoration in neurodegenerative disease – a preliminary investigation, by J Jiang et al.**

**Audiometry procedure**

We followed the British Society of Audiology recommended procedure for pure-tone audiometry as per the 2018 guideline (BSA, 2018), using the dual-channel GSI Audiostar Pro (*GSI AUDIOSTAR PRO TM USER MANUAL*, 2013). All testing took place in a quiet room, and was administered via the calibrated GSI Audiostar Pro headphones, with calibrated, noise-reducing ear-cups provided by the supplier. Testing was commenced at the better hearing ear (according to each participant’s account) at 1000 Hz. Frequency testing proceeded as follows, 2000 Hz, 4000 Hz, 8000 Hz, 500 Hz and 250 Hz and for the first ear only, 1000 Hz was retested to ensure a 5 dB variation or less. Tone duration was between 1 and 3 seconds and the duration between tone presentations was varied between 1 and at least 3 seconds, avoiding predictability. Subjects were instructed to indicate if they could hear a tone by pressing a clicker.

Averages were generated across thresholds at 500, 1000, 2000 and 4000Hz. Where there was a discrepancy between ears, we took the score from the better ear, and this ‘peripheral hearing composite score’ was used as a measure of each participant’s general peripheral hearing.

**Details of experimental stimulus synthesis**

Forty tri-syllabic words with ‘target’ consonant *t, p, f, s, d* or *c* were chosen and separated into two lists each comprising 20 words, matched for phoneme, familiarity, concreteness, imageability, written frequency using the MRC Psycholinguistic database (<https://websites.psychology.uwa.edu.au/school/mrcdatabase/uwa_mrc.htm>; see Supplementary Table 1). Consonants rather than vowels were targeted for the noise manipulation, as consonants have been shown to produce stronger phonemic restoration effects in normal listeners due to their acoustic similarity to noise (Samuel, 1981). Forty matched pseudowords were created by changing specific phonemes in each of the real words, generating new but phonetically plausible pseudowords (e.g., the real word “history” became “bistoty”; Supplementary Table 1), such that the ‘target’ consonant remained unchanged in each case. The target consonant was never the initial or final phoneme of the word.

Recordings were made of each real word and pseudoword being read aloud by a male native English speaker with a Standard Southern British English accent on a JoeMeek JM47a Meekrophone on a 2013 Macbook Air. Audio Software utilised were the Scarlett 2i2 First Generation Audio Interface and Reaper digital audio workstation (DAW).

Recordings were then edited using Praat software (<https://www.fon.hum.uva.nl/praat/>) to generate stimuli in which the target consonant was altered by insertion of a white noise segment, such that the segment either replaced the native consonant or was added to it (following del Tufo and Myers, 2014). The white noise segments were created in Praat, setting the formula to randomGauss(0,0.25). In each case, the segment containing white noise was of equivalent duration and mean power to the original phoneme. Spectrograms of representative stimuli are shown in Figure 1.

**Rationale for using A’**

Previous studies of phonemic restoration have used signal detection theory, specifically ***d’*** as a measure of the sensitivity of restoration (Samuel, 1981; Del Tufo and Myers, 2014). In this context, ***d’*** reflects the discriminability of ‘Added’ and ‘Replaced’ versions of the same word. However, upon inspection of our data (see Table 3), it became apparent that individual participants across the healthy control and patient groups never mislabelled an ‘Added’ stimulus as ‘Replaced’ in the noise segment and real word conditions, giving a value of 0 for this response cell and rendering the use of d’ untenable. For this reason, we opted instead to use the nonparametric ***A’***, which has similar properties to **d’** (Stanislaw and Todorov, 1999).

**Supplementary Table 1.** Words and pseudowords used in phonemic restoration experiment

| **Real words** | | | **Pseudowords** | | | **Manner** | **Placement** |
| --- | --- | --- | --- | --- | --- | --- | --- |
|  | **A\|A** | **R\|A** |  | **A\|A** | **R\|A** |  |  |
| A/PP/EARANCE | 21,4,5 | 1,0,3 | I/PP/EAGANCE | 12,3,1 | 4,0,0 | Stop | Early |
| ASSI/S/TANCE | 22,4,5 | 1,0,2 | ABBI/S/TINCE | 12,3,0 | 0,0,1 | Fricative | Mid |
| ATMO/S/PHERE | 21,4,4 | 9,1,4 | ALMO/S/BERE | 11,4,1 | 3,1,1 | Fricative | Mid |
| ATTI/T/UDE | 22,4,5 | 3,1,3 | AFFI/T/UGE | 13,4,0 | 2,0,0 | Stop | Late |
| CA/P/ITAL | 21,4,5 | 22,4,5 | HA/P/IFAL | 12,4,0 | 7,2,2 | Stop | Mid |
| CEN/T/URY | 20,4,5 | 7,2,1 | CIN/T/URAB | 18,4,1 | 5,1,1 | Stop | Mid |
| CHARAC/T/ER | 22,4,5 | 19,4,5 | RARAC/T/ED | 19,4,1, | 8,2,0 | Stop | Late |
| COM/P/ANY | 21,4,4 | 19,4,5 | DOM/P/ANED | 18,4,0 | 17,4,1 | Stop | Mid |
| CON/D/ITION | 22,4,4 | 1,0,1 | BON/D/ILON | 17,4,0 | 8,2,0 | Stop | Mid |
| CON/F/IDENCE | 22,4,5 | 9,1,3 | PON/F/IDENG | 18,4,1 | 4,0,0 | Fricative | Mid |
| CON/F/USION | 20,4,5 | 22,4,5 | FON/F/URON | 18,4,1 | 18,4,1 | Fricative | Mid |
| CONS/T/RUCTION | 21,4,4 | 16,3,5 | DONS/T/RUCFEN | 16,4,0 | 7,3,1 | Stop | Mid |
| DE/C/ISION | 22,4,5 | 19,4,5 | BE/C/IDON | 17,4,1 | 11,4,1 | Fricative | Mid |
| DEPAR/T/MENT | 21,4,5 | 12,0,1 | GEPAR/T/FENT | 11,3,0 | 5,1,0 | Stop | Mid |
| DESCRI/P/TION | 22,4,5 | 18,2,5 | DEFRI/P/BON | 13,3,1 | 13,3,1 | Stop | Mid |
| EM/P/LOYMENT | 18,3,2 | 3,1,3 | ED/P/LOFMENT | 7,4,0 | 1,0,0 | Stop | Mid |
| ENTER/P/RISE | 22,4,5 | 7,0,2 | ENFER/P/RASE | 13,4,0 | 1,0,0 | Stop | Late |
| EQUI/P/MENT | 22,4,4 | 2,0,1 | EBI/P/LENT | 11,3,0 | 0,0,1 | Stop | Mid |
| EX/P/RESSION | 16,3,4 | 1,0,2 | UX/P/REDDON | 12,3,1 | 3,0,0 | Stop | Mid |
| HIS/T/ORY | 22,4,5 | 20,4,5 | BIS/T/OTY | 13,4,0 | 5,1,0 | Stop | Mid |
| HOSPI/T/AL | 20,4,5 | 21,4,5 | HISPI/T/AD | 19,4,1 | 20,4,0 | Stop | Late |
| IMPOR/T/ANCE | 20,4,5 | 21,3,5 | AMPOR/T/ANE | 19,3,1 | 6,2,2 | Stop | Late |
| INDU/S/TRY | 20,4,4 | 1,0,0 | ILDU/S/TAY | 17,4,2 | 1,0,0 | Fricative | Mid |
| IN/S/TITUTE | 20,4,5 | 18,1,4 | IB/S/TITITE | 11,3,1 | 8,1,0 | Fricative | Early |
| MINI/S/TER | 22,4,4 | 20,4,5 | MUNI/S/GER | 18,4,0 | 14,3,0 | Fricative | Mid |
| NEW/S/PAPER | 19,4,5 | 3,1,2 | HEW/S/PADER | 14,4,1 | 3,0,1 | Fricative | Early |
| O/FF/ICER | 21,4,5 | 5,0,2 | U/FF/IYER | 5,3,1 | 0,0,0 | Fricative | Early |
| O/P/INION | 21,3,5 | 12,4,4 | U/P/IDION | 20,4,0 | 2,0,1 | Stop | Early |
| ORCHES/T/RA | 22,4,5 | 21,3,5 | ORFES/T/RID | 15,4,1 | 7,2,2 | Stop | Late |
| PERCE/P/TION | 22,4,5 | 22,4,5 | LERCE/P/RON | 15,3,1 | 5,1,2 | Fricative | Mid |
| PER/S/ONNEL | 22,4,5 | 1,1,3 | POR/S/OBBEL | 14,4,1 | 5,0,0 | Stop | Mid |
| POE/T/RY | 22,4,5 | 16,2,5 | HOE/T/ID | 18,4,0 | 5,2,0 | Stop | Late |
| PRINCI/P/LE | 22,4,5 | 21,4,5 | GRINCI/P/IT | 18,4,1 | 9,3,0 | Stop | Late |
| PRO/D/UCTION | 21,4,4 | 19,4,5 | PLO/D/UCFON | 10,3,1 | 6,2,1 | Stop | Mid |
| PRO/F/ESSOR | 22,4,5 | 6,0,4 | TRO/F/ETTOR | 14,3,2 | 2,0,0 | Fricative | Mid |
| PRO/P/ERTY | 22,4,5 | 17,2,4 | GRO/P/ERFY | 18,4,1 | 16,3,1 | Stop | Mid |
| PRO/T/ECTION | 20,4,5 | 4,0,0 | FRO/T/ECTAN | 12,3,2 | 0,0,1 | Stop | Mid |
| RA/D/IO | 22,4,5 | 10,2,5 | BA/D/IA | 18,4,1 | 9,1,0 | Stop | Mid |
| RESIS/T/ANCE | 17,4,2 | 10,4,0 | BESIS/T/ANG | 22,4,5 | 22,4,5 | Stop | Late |
| TEN/D/ENCY | 22,4,5 | 2,0,2 | REN/D/ENFY | 17,3,1 | 5,0,0 | Stop | Mid |

Pseudowords in each case were generated by modifying specific phonemes of the ‘matching’ real word. The ‘target’ phoneme that underwent noise modification in each word stimulus is framed by / /. For each stimulus, the ‘A|A’ column denotes the number of participants in each group (in order: control, SD, AD) who correctly identified the ‘Added’ version as ‘Added’; ‘R|A’ gives the number of participants in each group who incorrectly identified the ‘Replaced’ version of each stimulus as ‘Added’ (i.e., perceptually restored the word). The column headed ‘Manner’ indicates the manner of articulation of the target phoneme, and ‘Placement’ refers to the part of the word in which the target phoneme was located: ‘early’ if it occurred in the first syllable; ‘mid’ if in the second; and ‘late’ if in the third. A two-tailed t-test showed that real and pseudowords did not differ significantly in target phoneme mean duration (real words, mean 105 (standard deviation 21) msec; pseudowords 105 (22) msec; p = 0.99).

**
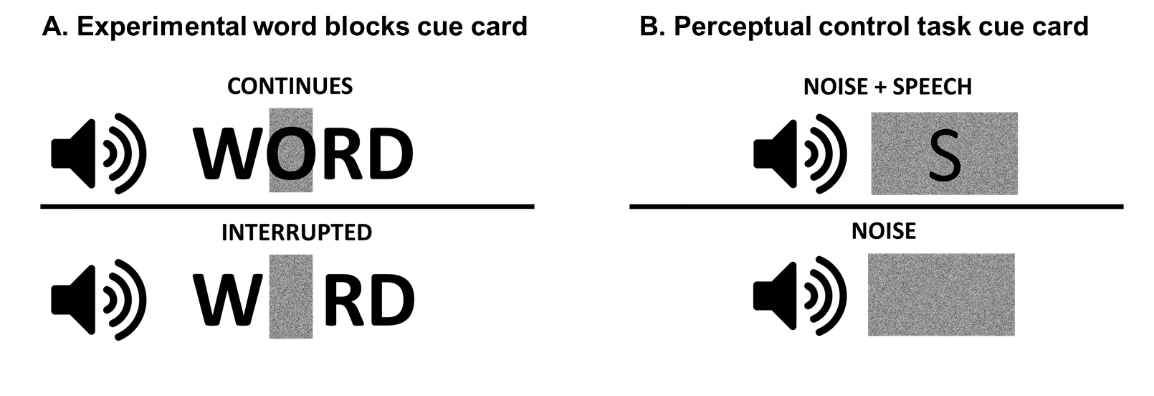
**

**Supplementary Figure 1.** Cue-cards used to help participants respond during the experiment.
